# Overcoming Barriers to Dental Care in India by the Use of Mobile Dental Vans

**DOI:** 10.7759/cureus.47786

**Published:** 2023-10-27

**Authors:** Hussain Ali John, Amit Reche, Priyanka Paul

**Affiliations:** 1 Public Health Dentistry, Sharad Pawar Dental College and Hospital, Datta Meghe Institute of Higher Education and Research (Deemed to Be University), Wardha, IND

**Keywords:** mobile health units, dental vans, portable dentistry, mobile dentistry, mobile dental vans

## Abstract

The aim of this literature review was to compile and present information on the use of mobile dental vans (MDVs) in India and to figure out if the use of these vans can be used to eliminate the barriers that prevent Indian people from getting access to quality and affordable dental care. Since the working of an MDV is quite complex, this article also serves to summarize the information available in the existing literature in a much simpler yet elaborate manner.

An electronic database search was done using relevant keywords such as MDVs, mobile health units, dental vans, mobile dentistry, and portable dentistry on search engines PubMed, Scopus, and Medline. After removing the irrelevant and duplicate articles, 30 were shortlisted and reviewed.

It is a difficult task to provide access to affordable dental care to every person in a country like India, which has such a large population and a large number of people living in rural areas. People who live in rural areas and/or belong to a lower socioeconomic class are more susceptible to oral diseases. Lower economic status, geographical location, motivational barriers, lack of public transportation, etc., deter people from receiving adequate dental care. MDVs are vehicles that can work as independent, efficient operatories to provide dental care and act as an adjunct as well as an alternative to conventional methods of improving the oral health of the community.

The global impact of MDVs, coupled with efficient and organized implementation, can make them an instrumental tool for the good. The various barriers to providing dental care to the underserved groups of the population can be easily overcome through this powerful tool.

## Introduction and background

In the world we live in today, we are connected more than ever, socially as well as physically, but there remain areas in developed as well as developing countries where people do not have adequate access to dental care. Due to this, they are unable to attain proper oral healthcare for themselves and their families. People living in rural areas usually belong to the poorer sections of society and cannot afford dental treatment. People of this economic class usually neglect their oral well-being, which leads to the further deterioration of their oral health. If the disease is not addressed at an early stage, the lesion or disease can progress to an advanced stage, and the cost of the treatment would be much higher than it was earlier. Since they can’t afford these higher costs, the absence of intervention might lead to complications like the development of systemic diseases and conditions and even morbidity [1]. If oral health care is to be accessible to all, it must be affordable and available to all sections of society irrespective of age, economic status, and geographical location. Special attention also needs to be paid to people suffering from physical and mental conditions that restrict them from commuting long distances. There are various ways of providing oral care to people. However, the goal is the same, which is to eliminate the disease or reduce the detrimental effects of the diseases that plague society [2,3]. Mobile dental vans (MDVs) or mobile dental units (MDUs) are vehicles that are fully equipped to render dental care to patients. The Indiana Administrative Code defines a "Mobile Dental Facility" as “any self-contained facility in which dentistry will be practiced, which may be moved, towed or transported from one location to another” [4]. They can be a boon to underserved sections of society that do not have adequate access to dental services. MDVs can be heavy motor vehicles like trucks or buses, which have been modified to hold various equipment necessary to provide essential dental care. They can be powerful tools in overcoming barriers in administering dental care [4,5]. However, the treatment procedures that can be performed at an MDV are limited. A practitioner might not be able to perform advanced screening and treatment modalities like complex oral surgeries as the equipment required for such procedures is unavailable in these vehicles [6-9].

MDVs have been viewed as an alternative as well as an adjunct to the conventional ways of providing dental care to the public. They have been preferred by hospitals because of their high mobility and lower costs when compared to the cost of setting up a brand-new establishment [10]. They can act as the first form of exposure to educate people about oral health. MDVs can be effectively employed for community outreach programs, community training, and rural postings of interns. Postgraduate students can also be employed to bolster the manpower required in such programs. In return, such students can gain valuable experience [11,12]. A major disadvantage of MDVs is the upfront cost of such vehicles and the cost of their maintenance. Every individual practitioner cannot afford to buy and maintain an MDV. Hence, it is usually observed that such vehicles are owned by dental colleges or multi-specialty dental hospitals [5].

## Review

Barriers to providing dental care

Many barriers prevent providing efficient dental care to the masses; they are:

Economic Barriers

Affordability is one of the significant issues that has plagued dentistry for a long time. People tend to avoid going to their local dentist because of economic constraints. Hence, the priority towards oral health among people of lower socioeconomic classes is very low [13]. Because of this, the dental disease can worsen, leading to a poorer prognosis and also might lead to complications along with an increase in treatment costs.

Geographical Barriers

Rural communities, when compared to communities living in urban areas, generally have fewer resources and less access to healthcare facilities due to their geographical location. The number of doctors providing dental care in rural areas is few; this is because it is difficult to hire and keep practitioners employed in such a location with few prospects. Even if dental clinics and personnel are present, the supply of water, electricity, and equipment is often unreliable. Hence, people living in rural areas are more susceptible to oral diseases due to low standards of dental care which may be available and are more likely to have a lower oral quality of life as compared to their urban counterparts [4,14,15].

Transportation Barriers

Isolated communities which are located far away from cities usually lack access to dental services. Even if patients are willing to travel, the lack of a public transport system can pose a threat to their goal. Lack of proper roads and not well-connected areas also are a hindrance. The natural topography of the area might also restrict access to vehicles, bringing help as it is difficult to traverse dense forests and areas of high altitude [4,16].

Having a Chronic/Debilitating Illness

People suffering from physical and mental diseases and conditions are often ill-suited for long commutes. Patients suffering from conditions that restrict their mobility, differently abled patients often are unable to get proper dental care. Many conditions like muscle dystrophy, neuromuscular impairments, rheumatoid arthritis, obesity, and old age may become a barrier for a person seeking oral care [5,17,18].

Lack of Awareness as a Barrier

Inadequate awareness about dental health and dental care might lead to people not opting to visit their local dentist and tend to ignore their oral health even if they are suffering for a long period. This is the most crucial barrier to overcome; however, this can be done through public health programs and combined efforts by private and public organizations [12].

Working on an MDV

MDVs are self-sufficient units in which equipment required for diagnosis and treatment is contained in a vehicle that is also meant for transportation [6]. The vehicle consists of various compartments that serve their function. It is the collaboration of all these compartments that makes MDVs a powerful tool for community outreach programs. The various compartments are a driving compartment and an area for the dental chair with provision for fresh water and recycling. There is also a place where registration of the patients can be done to maintain a record for legal and follow-up purposes. An MDV might also have a surgery room, which is utilized for certain procedures [5].

**Figure 1:**
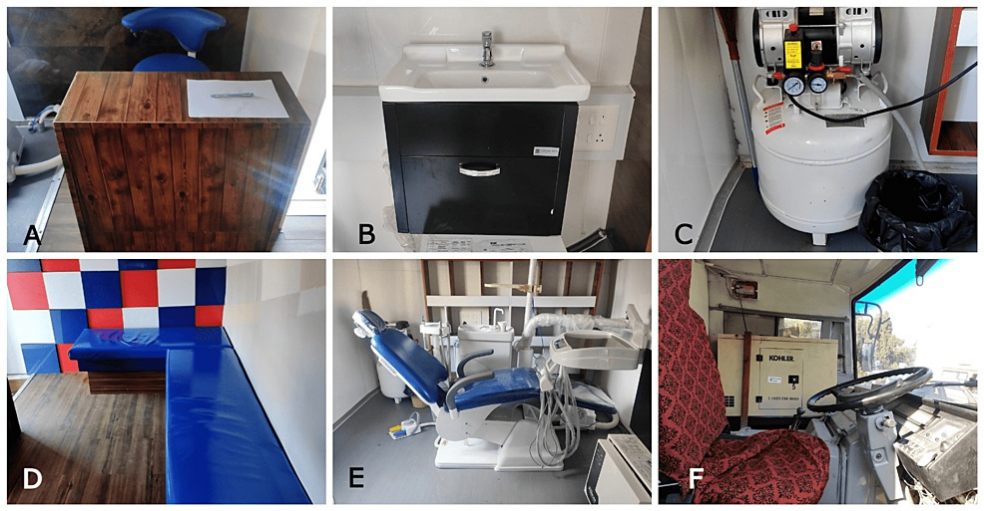
Various sections and compartments in a mobile dental van (A): Registration area; (B): Sink area; (C): Area for compressor; (D): Patient waiting area; (E): Area for dental chair; (F): generator and driving area. The images have been taken by the author with consent from the hospital which owns this mobile dental unit.

The systems and equipment that an MDV contains can be simple or complex. A simple system contains only the basic fundamental equipment, including mouth mirrors, probes, and tweezers. Apart from this, it might also include flashlights and personal protective equipment, all organized in cases. Complex systems might have an elaborate and vast number of items and equipment like a dental chair, ultrasonic scalers, handpieces of different speeds, materials, and instruments for restorations, extractions, and preventive procedures. Radiography equipment is also carried. All this requires a bigger and much more durable case [17,19]. Figure 1 shows the sections and compartments of the MDV.

An experienced maintenance team is essential to repair the vehicle and the equipment within the various compartments if and when they break down. The team must have adequate knowledge about the mechanical, electrical, and biomedical aspects of the vehicle and the equipment [5].

Scope of MDVs in India

As of 2023, India has the second-highest population in the world, with more than 1.4 billion people. The vast majority of this population lives in rural or semi-rural areas which lack basic dental services. In developing countries like India, oral diseases are a massive burden for people living in rural areas [4,20]. A rapid increase in the number of oral diseases has been observed in the past 10-20 years. Also, the use of deleterious products like tobacco and betel nut has contributed to India being called the "Oral cancer capital" of the world [21].

India has a poor dentist-to-population ratio of about 1:30000. The World Health Organization recommends that this ratio should be 1:7500 to achieve optimal oral health. More than four-fifths of the dental practitioners in India work in major cities. To solve this disparity, at least 31% of practitioners must work in rural areas [22]. The mode of payment in urban areas is mostly private fee for service. This is not ideal for patients with lower socioeconomic status as they can’t afford the high costs of treatment [23]. MDVs offer an innovative solution to these problems, and the various applications of MDVs may help overcome the barriers to providing dental care to every person [3].

Applications of MDVs

Improvement of Community Health

MDVs are effective in tackling many barriers to dental care. MDVs are being employed for postings of interns and postgraduate students in rural areas and treatment camps in various colleges across India. Many different procedures can be carried out in such vehicles, such as oral prophylaxis, application of pit and fissure sealants, fluoride application, restorations, and simple extractions [4]. Apart from carrying out treatment procedures, various screening services can also be imparted. Screening of oral diseases, as well as systemic diseases like hypertension and diabetes mellitus, can also be performed along with immunization of children and high-risk patients [24].

Health Education at Schools and Villages

Medical and dental colleges have a significant part in imparting healthcare and health education to underserved populations. MDVs can help carry out programs in various communities and institutions and educate them about the importance of oral health and the treatment options available [25].

Boon for the Underserved and Isolated Populations

MDVs can be a catalyst in eliminating rural-urban inequality in terms of dental care. The underserved populations like rural communities, people belonging to lower socioeconomic classes, children, the homeless, and migrant populations can also benefit immensely from the correct utilization of these vehicles. According to Mulligan et al., the need for dental intervention in migrant children is higher. Thus, these vans can be instrumental for their oral health [26]. One of the striking features of these vehicles is their mobility and transport. As all areas are not accessible by a conventional vehicle, MDVs provide a unique solution as they can access areas that can’t be traversed and are isolated due to natural topography, like high altitude or dense forests [16].

Improve No-Show Rates

It has been observed children and aged people belonging to poverty-stricken families are more susceptible to oral diseases and also have higher no-show rates. These groups usually have a lack of access to care and transportation because they are at the behest of their caregiver. Even if a child has dental insurance, the parents are not motivated to take their child to a dental practitioner. MDVs have the potential to reduce missed appointments, especially when programs are run in coordination with the local schools. MDVs can be revolutionary in solving this problem as doorstep delivery of dental service is one of its key features [3,27].

Utilization in Internship Program

In India, the undergraduate degree for dentistry is called Bachelor of Dental Surgery. It is awarded to a student after five years, which includes one year of mandatory internship. Interns or newly graduated dentists are not very oriented to community health and are not able to understand the significance of their role as caregivers and translators of scientific knowledge to common people. As MDVs are a powerful tool in carrying out health education and community health programs, they can also be used to educate interns and new dentists associated with the colleges and hospitals of the country [28].

Greater overall impact than an independent dental practitioner

It is very difficult for an independent dental practitioner to impact the community oral health of a population on a large scale, while it is possible to do so with the help of a mobile dental can. The differences between the two are mentioned in Table 1.

**Table 1:** Differences between a mobile dental van and an independent dental clinic [5-9,16,19,25,29,30]

Mobile dental van	Independent dental clinic
It is accessible to a larger population	It Is accessible to a limited number of people
The cost to set up is lesser	The cost to set up is higher
The equipment required is less expensive	The equipment required is usually more expensive
The cost and frequency of maintenance are higher	The cost and frequency of maintenance are lower
Limited treatment and screening procedures can be performed	A wide variety of treatment and screening procedures can be performed
Availability is impacted by natural causes like weather and the topography of the area	Availability is not impacted by natural causes
Does not require the practitioner to stay at the MDV at all times	Requires the practitioner to stay at the clinic at all times
It can act as a channel between dental practitioners and the underserved populations of society	It cannot act as a channel or serve the underserved populations of society

Limitations/disadvantages of MDVs

Although MDVs are a powerful tool in overcoming several barriers to availability and accessibility to dental care, they have limitations and disadvantages as well. Table 2 shows the applications and limitations of mobile dental vans.

**Table 2:** Applications and limitations of mobile dental vans [4,5,9,16,24-26,29,30]

Applications	Limitations
Helps improve the oral health of the community	High cost of maintenance
Provides health education at various institutions and communities like schools and villages	Advanced treatment and screening procedures cannot be performed
It is an instrumental tool for providing dental care to the underserved population of society	The weather might impact its availability
Improves no-show rates for dependent populations like children and the elderly	Not meant to cover long distances
Training of interns and newly graduated dentists	Requires specialists to fix broken equipment and machinery

High Costs of Maintenance

MDVs have a substantially high cost of maintenance, the initial cost of a vehicle might be less than setting up a dental clinic from scratch, but the maintenance costs are higher. This is because an MDV is nothing but a complex machine with many parts and compartments. The maintenance fee is high, and the parts to be repaired or replaced are expensive [29].

Limited Procedures Can Be Performed

Not every treatment procedure can be performed at an MDV; complex and highly invasive procedures require a large number of instruments, constant monitoring, and an environment with strict sterile conditions. All this is not possible in an MDV. Most units do not have laboratories that are required for fabricating various prostheses [9,30].

Limited Access

This is one of the major drawbacks of the MDVs; although these vans have great mobility, they are also not meant to be transported for long distances. Hence, they can’t be used to provide dental services to communities that are located very far away. MDVs are heavy motor vehicles and are not suitable for all types of terrain; therefore, their use is quite limited when it comes to hilly areas, areas with marshy soil, and areas poorly connected by roads [8].

Impact of the Weather

The availability of an MDV is impacted by weather conditions. Certain areas and roads might be blocked off or inaccessible due to unfavorable weather conditions limiting the use of such vehicles. The meteorological conditions of an area may stem from very wet to very dry and everything in between, and it is not possible for a vehicle like an MDV to traverse through such rough areas without damaging the internal apparatus [5].

## Conclusions

Every person in the world deserves access to affordable dental care. However, there are several hindrances in achieving this goal. These hindrances or barriers can be classified as economic, transportation, geographical, etc. To overcome these barriers a combined effort by individuals, healthcare societies, and the public is required, and only then can a comprehensive plan be formulated. A unique solution to these problems can be the use of MDVs. These vans can overcome a majority of these barriers at a fraction of the cost when compared to the cost of setting up big hospitals in many locations. To provide quality dental care in a country like India which has a huge population, we need to think of unique ways to tackle this problem; hence, MDVs with their superior mobility and utility can help serve even the most underserved people of the country.
